# Natural products for managing metabolic syndrome: a scoping review

**DOI:** 10.3389/fphar.2024.1366946

**Published:** 2024-04-30

**Authors:** Mohammed Faris Abdulghani, Sadeq Al-Fayyadh

**Affiliations:** Nursing College, University of Baghdad, Baghdad, Iraq

**Keywords:** natural products, plants, complementary and alternative medicine, metabolic syndrome, scoping review

## Abstract

**Introduction:**

Metabolic syndrome comprises a collection of metabolic disorders stemming from factors like genetic predisposition, inadequate nutrition, stress, decreased physical activity, aging, and ethnicity. Although traditional pharmaceutical treatments exist for metabolic syndrome, their limited popularity is attributed to high costs and adverse effects. Consequently, natural products with fewer side effects have been explored for managing this condition. This literature review aims to explore the role of natural products including herbs, botanicals, vitamins, minerals, probiotics, and dietary supplements in managing metabolic syndrome.

**Methods:**

This scoping review was conducted in five steps, involving the formulation of a research question, the retrieval and extraction of relevant studies, the selection of pertinent studies, the organization of information into tables, and the reporting of results. Data was collected from various databases including Embase, Science Direct, PubMed, Google Scholar, Scopus, and Web of Science, with a focus on studies published from 2010 to the present, available in English and with full-text accessibility.

**Results:**

We identified 1,259 articles, screened their titles, abstracts, and full texts, ultimately incorporating 169 pertinent articles into this review (comprising 90 review articles, 32 trial articles, 6 *in vitro* articles, 38 *in vivo* articles, 1 experimental article and 2 observational articles). The study’s outcomes revealed that natural products, encompassing plants and their derivatives, vitamins and supplements, as well as probiotics, can exert a beneficial influence on metabolic syndrome by regulating blood sugar, blood pressure, lipid profiles, obesity, and abnormal cholesterol and triglyceride levels.

**Conclusion:**

The current study underscores the significance of natural products in addressing metabolic syndrome. Consequently, it is advisable to conduct further extensive research to assess the efficacy of these products, potentially integrating them into treatment regimens for individuals with metabolic syndrome.

## 1 Introduction

Metabolic syndrome (MetS) is a cluster of metabolic disorders, including impairments in protein, glucose, lipid, and carbohydrate (Di et al., 2019). It is characterized by a combination of risk factors for atherosclerotic cardiovascular diseases (ASCVD) and type 2 diabetes (Hou et al., 2019; Nna et al., 2023). These risk factors lead to the development of high blood pressure, high blood sugar or diabetes, obesity, excess abdominal fat, and abnormal cholesterol or triglyceride levels (Hou et al., 2019). The diagnosis of metabolic syndrome typically requires the presence of three or more of the following conditions: (Di et al., 2019): waist circumference ≥102 cm in men and ≥88 cm in women, (Nna et al., 2023), triglycerides ≥150 mg/dL or undergoing drug therapy for high triglycerides, (Hou et al., 2019), HDL-C < 40 mg/dL in men and <50 mg/dL in women or undergoing drug therapy for lowering cholesterol, (Jang et al., 2016a), systolic blood pressure ≥130 mmHg, diastolic blood pressure ≥85 mmHg or undergoing antihypertensive treatment for individuals with a history of hypertension, and (van der Pal et al., 2018) fasting glucose ≥100 mg/dL or undergoing treatment to control glucose levels (Jang et al., 2016a; van der Pal et al., 2018). However, the specific measurement for waist size may vary slightly based on country and ethnicity (Jang et al., 2016a). Factors contributing to the development of metabolic syndrome include genetic predisposition, poor dietary habits, stress, sedentary lifestyle, age, and ethnicity (Raja Kumar et al., 2019; Nna et al., 2023). Some of these factors, such as diet and physical activity, can be managed over time (Raja Kumar et al., 2019).

The treatment of metabolic syndrome has traditionally involved dietary modifications and the use of chemical drugs targeting specific biochemical pathways involved in food metabolism (Hameed and Al-Ameri, 2022). Beta-blockers, statins, fibrates and glibenclamide are usually known as the most commonly used drugs for patients with metabolic syndrome (Gharipour et al., 2011). However, these drugs are often costly, poorly tolerated by patients, associated with various side effects and do not have sustainable effectiveness (Nigussie, 2021). Additionally, typically represent monotherapy and address only a limited range of health outcomes associated with metabolic disorders (Tola et al., 2023). Consequently, there is a need to explore and develop alternative and complementary approaches with fewer complications for managing metabolic diseases (Nigussie, 2021).

Complementary and alternative medicine (CAM) has been used globally for centuries and consists of a wide range of therapies (Suganya et al., 2017; Adeniyi et al., 2021; Nasiri et al., 2023). One of these treatments is the use of natural products (Nasiri et al., 2023). Many medicinal plants and natural products are considered by the public as a safe and natural alternative to synthetic drugs (Waltenberger et al., 2016b). It has been shown that natural products or their derivatives are a valuable source of therapeutic agents (Atanasov et al., 2015; Fadhel and Hassan, 2023). Growing evidence indicates that natural products and their bioactive compounds can provide various benefits to the human health (Nainu et al., 2023). Natural products such as herbal drugs in addition to their secondary metabolites act as endless sources of promising drug leads that revealed significant anti-inflammatory as well as anti-obesity potential (Youssef et al., 2022). They revealed higher safety margins, eco-friendly, and less expensive with respect to synthetic chemical entities (Li et al., 2021). Consistent with this approach, researchers have focused on natural products in the field of prevention or treatment of MetS (Dong et al., 2012; Taghipour et al., 2019). In this regard, a study by Jang et al. (2016) demonstrated the effect of herbal medicines on reducing waist circumference, blood glucose, blood lipids, and blood pressure, suggesting their potential for treating metabolic syndrome (Jang et al., 2016a). Sabarathinam et al. (2022) highlighted that certain plant extracts contain natural active components that target multiple biological pathways, offering the opportunity to address various defects associated with metabolic syndrome simultaneously (Sabarathinam et al., 2022). Additionally, a study by Taghipour et al. (2019) revealed that Plant-based natural products improve obesity-associated MetS such as hyperglycemia, hyperlipidemia, and insulin resistance, reduce systolic and diastolic blood pressure, and reduce body weight gain (Taghipour et al., 2019).

Metabolic syndrome is now recognized as a disease with developmental origins, and it has become a significant focus of recent research (Hou et al., 2019). Given the increasing popularity of complementary and alternative medicine (CAM) for managing various health conditions, including metabolic syndrome, a review of the existing literature is crucial to comprehend the scope of CAM usage, its reported effectiveness, and potential safety considerations in relation to metabolic syndrome. While there is evidence suggesting that certain natural products can positively affect components of metabolic syndrome (Jang et al., 2016a; Taghipour et al., 2019; Sabarathinam et al., 2022), a comprehensive review is necessary to evaluate the quality of this evidence. Moreover, there are still gaps in understanding which natural products show the most promising outcomes, their mechanisms of action, and their potential interactions with conventional therapies. A scoping review can help identify these gaps and guide the design of future studies.

Healthcare practitioners and patients require evidence-based information to make informed decisions about the use of natural products for managing metabolic syndrome. Therefore, conducting a scoping review can offer a comprehensive overview of the current state of knowledge and help elucidate the potential role of natural products in this field. This scoping review aimed to investigate natural products in the management of metabolic syndrome.

## 2 Materials and methods

This scoping review was conducted in five stages: 1) formulating the research question, 2) searching for and extracting relevant studies, 3) selecting related studies, 4) tabulating, summarizing, and synthesizing the information and data, and 5) reporting the results (Mak and Thomas, 2022). Following the formulation of the research question (what is the role of natural products in the management of patients with metabolic syndrome?), a search strategy was devised, inclusion criteria for the selected studies were established, data extraction forms were prepared, and data analysis program was specified.

### 2.1 Information sources and searches

In the book authored by Pinzon-Perez et al. (2015) (Pinzon-Perez and Pérez, 2016), 6 sub-categories for natural products (herbs, botanicals, vitamins, minerals, probiotics, and dietary supplements) are delineated, and the researchers also employed this keywords to devise an optimal search strategy. For this purpose, researchers conducted searches across several databases, including Embase, Science Direct, PubMed, Google Scholar, Scopus, and Web of Science. They used a range of keywords such as “herbs”, “botanicals”, “vitamins”, “minerals”, “probiotics”, “dietary supplements”, “natural product”, “essential oils” and “metabolic syndrome” to retrieve information from these databases.

### 2.2 Inclusion and exclusion criteria

The inclusion criteria included reviewing all experimental, quasi-experimental, systematic and Meta-analysis, clinical trial, review, interventional, observational, *in vivo* and *in vitro* articles, A full texts articles which were published in reputable journals were included. Additionally, articles related to animal phases were considered. The exclusion criteria covered articles that did not specifically investigate the impact of complementary and alternative medicine on metabolic syndrome, studies published before 2010, and studies published in languages other than English.

### 2.3 Selection of relevant studies

**FIGURE 1:**
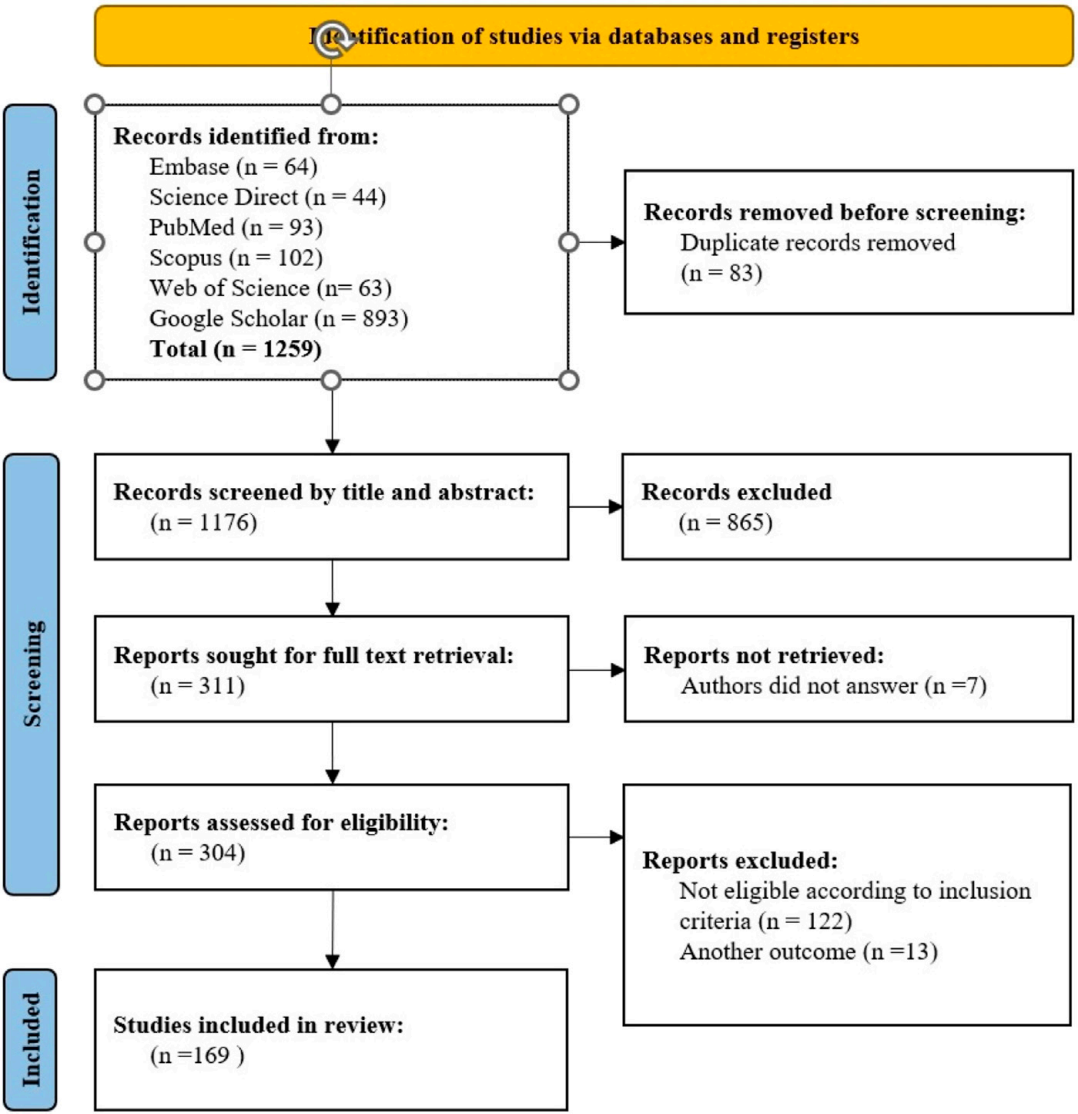
Flowchart of review and selection of articles.

Initially, one of the researchers imported all search results from the databases into the EndNote Desktop program, with duplicates removed. Subsequently, two researchers independently reviewed articles titles and abstracts based on predetermined eligibility criteria. Any discrepancies in study selection between the two researchers were resolved through a full-text evaluation. Efforts were made to obtain inaccessible articles and unpublished data by contacting the corresponding authors of eligible studies. Initially, 1,256 articles were identified through the database search. Following the elimination of 83 duplicates, 1,176 titles and abstracts were screened, leading to the review of 311 full texts. All of them underwent assessment for eligibility criteria culminating in the inclusion of 169 articles in the study (see Figure 1). Furthermore, the reference lists of the extracted articles were examined, but no additional articles meeting the inclusion criteria were identified for this study.

Data extraction and synthesis were carried out using a standardized form, which included categories such as study identifiers (study’s author, year of publication), country and language, study type, study objective, sample size, materials and methods, results, and conclusion(s).

To comprehensively understand the research landscape on natural products for the treatment of metabolic syndrome (MetS), researchers have adopted a systematic approach to categorize studies based on their methodology and focus. This classification includes clinical trials, *in vitro* and *in vivo* studies, observational studies, and review studies. Clinical trials provide empirical evidence on the efficacy and safety of interventions in human subjects, while *in vitro* and *in vivo* studies offer mechanistic insights into the effects of natural products on metabolic pathways and physiological functions. Observational studies identify associations between product consumption, natural outcomes related to metabolic syndrome, and contributing populations. These studies are referred to as original studies (see Table 1). Additionally, review studies provide a synthesis of the existing literature and highlight the importance of herbs and natural products in addressing MetS; therefore, they are considered complementary studies. Studies are categorized based on the type of natural product investigated, including herbs, vitamins, minerals, probiotics, and dietary supplements.

**TABLE 1:** Studies conducted on the effect of different natural products on metabolic syndrome.

Author	Country	Study type	Study aim	Sample size	Materials and methods	Results	Article’s quality
Herbs							
Nikaein et al. (2016) (Nikaein et al., 2016)	Iran	Clinical trial	The Effects of *Satureja hortensis L.* Dried Leaves on Metabolic Syndrome Patients	N = 47	Daily intake 1 capsule (450 mg) for 10 weeks	Reducing in total cholesterol, low-density lipoprotein cholesterol (LDL-C), triglycerides (TG), diastolic blood pressure (DBP), and hs-CRP as well as an elevation in high-density lipoprotein cholesterol (HDL-C)	High
Seong et al. (2021) (Seong et al., 2021)	Korea	Clinical trial	The effect of Korean red *Panax ginseng* on metabolic syndrome	N = 60	Receiving KRG capsules (Korean red ginseng powder 100%, 6,000 mg/day) or placebo capsules (4,200 mg/day) for 8 weeks	Decreasing systolic blood pressure (SBP)	High
Klupp et al. (2016) (Klupp et al., 2016)	Australia	Clinical trial	The effect of *Ganoderma lucidum* on metabolic syndrome	N = 84	Daily intake of 3 g for 16 weeks	No effect of Ganoderma on hyperglycemia and cardiovascular risk factors	High
Amin et al. (2015) (Amin et al., 2015)	Pakistan	Clinical trial	The effect of simultaneous consumption of *Curcuma longa* and *Nigella* on metabolic syndrome	N = 250	Using black seeds (1.5 g per day), turmeric (2.4 g per day), and their combination (900 mg of black seeds and 1.5 g of turmeric) in different groups for 8 weeks	Reducing waist circumference, lipid profile, BMI, cholesterol, fasting blood glucose and C-reactive protein	High
Belcaro et al. (2013) (Belcaro et al., 2013)	Italy	Clinical trial	The effect of green phytosome on metabolic syndrome	N = 100	Use of 2 tablets (total equivalent of 300 mg per day) for 24 weeks	Improvement of weight, lipid profile and blood pressure	High
Devaraj et al. (2013) (Devaraj et al., 2013)	The U. S	Clinical trial	The effect of *Aloe vera* on people with metabolic syndrome	N = 45	Daily intake of 2 capsules of 500 mg for 8 weeks	Reduction of LDL-C level, glucose and fructosamine, reduction of HbA1c, fasting glucose and insulin	High
Najmi et al. (2013) (Najmi et al., 2013)	India	Clinical trial	Effect of herbal product *Nigella sativa* on metabolic syndrome	N = 90	Receiving 500 mg/day capsule of Nigella sativa for 8 weeks	Antihypertensive, reducing LDL-c	High
Mansouri et al. (2012) (Mansouri et al., 2012)	Iran	Clinical trial	Effect of *Anethum graveolens* (dill) on metabolic syndrome	N = 24	Receiving 600 mg per day for 3 months	Reducing triglyceride	High
Shah et al. (2012) (Shah et al., 2012)	Pakistan	Clinical trial	Effect of *Nigella sativa* on metabolic syndrome	N = 159	Receiving 250 mg twice daily for 6 weeks	Beneficial effects in fasting blood sugar, low density lipoproteins and high density lipoproteins, blood pressure, circumference of waist and serum triglyceride	Moderate
Mohtashami (2019) (Mohtashami, 2019)	Iran	Clinical trial	Effects of Bread with *Nigella Sativa* on Metabolic Syndrome	N = 51	Receiving daily a bread with *N. sativa* for 2 months	No effect on FBG, BP, weight, WC, and BMI.	High
Basu et al. (2010) (Basu et al., 2010)	Oklahoma	Clinical trial	Effect of Green Tea Supplementation on Metabolic Syndrome	N = 35	Receiving green tea (4 cups/d) or green tea extracts (2 capsules, 4 cups water/d) for 8 weeks	Reducing body weight, BMI, LDL-cholesterol and LDL/high-density lipoprotein (HDL)	High
					(Each cup of green tea provided approximately 110 mg of EGCG1/Each capsule provided approximately 230 mg EGCG)		
Jain et al. (2017) (Gupta Jain et al., 2017)	India	Clinical trial	The effect of oral *Cinnamomum zeylanicum* on metabolic syndrome	N = 116	Daily intake of cinnamon [6 capsules (3 g)] or wheat flour [6 capsules (2.5 g)] for 16 weeks	Reducing waist-hip ratio, blood pressure, serum total cholesterol, low-density lipoprotein cholesterol, serum triglycerides, and high-density lipoprotein cholesterol	High
Morovati et al. (2019) (Morovati et al., 2019)	Iran	Clinical trial	Effects of cumin (*Cuminum cyminum L*.) essential oil supplementation on metabolic syndrome	N = 56	Receiving 75 mg CuEO or placebo soft gel thrice daily for 8 weeks	Reducing diastolic blood pressure	High
						No effect on the others parameters	
Ghitea et al. (2021) (Ghitea et al., 2021)	The U.S	Clinical Trials	The effect of *Origanum vulgare L*. on Metabolic Syndrome	N = 106	Receiving 0.8 mL (2 drops) twice a day for 10 days	Antibacterial effect	High
Lopes Galeno et al. (2014) (Lopes et al., 2014)	Brazil	*In vitro*	The effect of extract from *Eugenia punicifolia* on Metabolic Syndrome	-	-	Antioxidant, reducing carbohydrate absorption rate	Moderate
Mopuri et al. (2018) (Mopuri et al., 2018)	India	*In vitro*	The effects of *Ficus carica* on metabolic syndrome	-	Using extract of Ficus carica	Antidiabetic and antiobesogenic	Moderate
Jakubczyk et al. (2021) (Jakubczyk et al., 2021)	Poland	*In vitro*	The Influence of *Hypericum perforatum L*. on Metabolic Syndrome	-	Preparation of cookies with the St. John’s wort (SJW) herb at 0.5 and 1 g/100 g	Higher content of bioactive compounds and antioxidant and anti-metabolic syndrome effects	Moderate
Kulabas et al. (2018) (Kulabas et al., 2018)	Turkey	*In vitro*	The effect of *Lavandula stoechas* on metabolic syndrome	-	Aqueous extract of Lavandula stoechas was extracted and analyzed	Preventing of insulin resistance and inflammation	Moderate
Cicolari et al. (2020) (Cicolari et al., 2020)	Italy	*In vitro*	The effect of Hydromethanolic Extracts from *Adansonia digitata L*. on Metabolic Syndrome	-	Using extracts of *A. digitata L*	Antihypertensive and antidyslipidemic	Moderate
Chae et al. (2022) (Chae et al., 2022)	The U.S	*In vitro*	Evaluate the Efficacy of *Aquilaria sinensis* Flower Extract against Metabolic Syndrome	-	Using extracts of Aquilaria sinensis Flower	Hyperlipidemia and hyperglycemia	Moderate
Dobhal et al. (2022) (Dobhal et al., 2022)	India	*In vivo*	The effect of Lemongrass (*Cymbopogon citratus*) on metabolic syndrome	N = 42	Lemongrass ethanolic extract and aqueous extract were prepared and lemongrass oil were given to the animals for 42 days	Reducing body weight, BMI, and fasting blood sugar normalizing serum insulin, insulin resistance, leptin and lipid profile	Moderate
Mayer et al. (2019) (Mayer et al., 2019)	France	*In vivo*	Preventive Effects of the Marine Microalga *Phaeodactylum tricornutum* Metabolic Syndrome	N = 18	Receiving diet supplemented with 12% of freeze-dried microalga P. tricornutum	Decreasing Body weight, fat mass, inflammatory markers, insulinemia, Plasma total cholesterol, triacylglycerols	Moderate
Jakovljevic et al. (2018) (Jakovljevic et al., 2018)	Serbia	*In vivo*	The effect of *Aronia melanocarpa* extract on metabolic syndrome in mice	N = 60	Daily intake of 0.45 mL/kg extract for 4 weeks	Lowering blood pressure, improving heart function and glucose tolerance, reducing liver pathological changes and oxidative stress	Moderate
Preez et al. (2021) (du Preez et al., 2021)	Australia	*In vivo*	The effect of Nannochloropsis oceanica as a Microalgal Food Intervention on Metabolic Syndrome	N = 48	Receiving a diet containing 5% N. oceanica for 8 weeks	Decreasing fat mass, source of essential amino acids and prebiotics	Moderate
Owis et al. (2017) (Owis et al., 2017)	Egypt	*In vivo*	The effect of Leaves of *Cordia boissieri* on metabolic syndrome	N = 100	Receiving 5 mg/kg/day Cordia boissieri for 4 weeks	Improving insulin sensitivity, glucose tolerance, kidney function, lipid profiles, and reduced oxidative stress and inflammation	Moderate
Reshidan et al. (2019) (Reshidan et al., 2019)	Malysia	*In vivo*	The effect of *Pandanus amaryllifolius* extract on metabolic syndrome in rats	N = 30	The administration of 10% plant leaf juice extract in the diets of rats within various groups over an 8-week period	Decreased BMI, fat cell size, blood pressure, fasting glucose, triglycerides, and high-density lipoprotein	Moderate
Singh et al. (2017) (Singh et al., 2017)	Canada	*In vivo*	The alcohol extract of North American ginseng (*Panax quinquefolius*) on metabolic syndrome	-	Receiving 200 mg/kg/100 μL of NAGE daily	Balance between glucose and fatty acid metabolism, lipoprotein secretion, and energy homeostasis	Moderate
Thomaz et al. (2022) (Thomaz et al., 2022)	Australia	*In vivo*	The effect of Wasabi (*Eutrema japonicum*) on Metabolic Syndrome	N = 48	Intake 5% wasabi powder for 16 weeks	Reducing blood pressure, obesity, inflammation and lipid deposition	Moderate
Jamshidi et al. (2018) (Jamshidi et al., 2018)	Iran	*In vivo*	The effect of Wild pistachio (*Pistacia atlantica mutica*) oil on metabolic syndrome	N = 72	Intake hull and kernel oils of WP (2 mL/kg/day) for 10 weeks	Preventing hyperglycemia, hypertriglyceridemia, hypercholesterolemia, inflammation and pancreatic secretory disorders	High
Wang et al. (2020) (Wang et al., 2020)	Taiwan	*In vivo*	The effect *Cinnamomum zeylanicum* and *Taiwanofungus camphoratus* on Metabolic	N = 48	Receiving different doses of CO and TC for 24 weeks	Improving abnormal blood glucose regulation and restore the balance of gut microbiota	Moderate
			Syndrome				
Vílchez et al. (2022) (Vílchez et al., 2022)	The United Kingdom	*In vivo*	The effect of GlucoMedix (*Stevia rebaudiana* and *Uncaria tomentosa*) on metabolic syndrome in rats	N = 40	Different groups used doses of 250, 1,000 and 2000 mg for 28 days	Reduced hyperglycemia, reduced lipid profile and blood pressure	Moderate
Alauddin et al. (2016) (Alauddin et al., 2016)	Japan	*In vivo*	Effect of fermented rice bran on metabolic syndrome in rats	N = 12	Use of 2 g/kg fermented and nonfermented rice bran in different groups	Lowering blood pressure, improving leptin disorder and increasing serum adiponectin levels, improving glucose tolerance and lipid profile	Moderate
Bhaswant et al. (2015) (Bhaswant et al., 2015)	Japan	*In vivo*	The effect of Green and Black *Elettaria cardamomum* on Metabolic Syndrome	N = 72	Receiving diet supplemented with 3% green or black cardamom	Black cardamom attenuated the signs of metabolic syndrome while green cardamom exacerbated adiposity, decreased liver function and worsened cardiovascular structure and function	Moderate
Chul Kho et al. (2016) (Kho et al., 2016)	South Korea	*In vivo*	The effect of red ginseng and *Fallopia multiflora* on metabolic syndrome in rats	N = 50	Daily intake of 100 and 300 mg by different groups for 8 weeks	Improving blood pressure, obesity, high lipid profile, vascular inflammation and insulin resistance	Moderate
Kuate et al. (2015) (Kuate et al., 2015)	Malysia	*In vivo*	The effect of *Tetrapleura tetraptera* spice on metabolic syndrome indicators	-	Using different oral doses of HET (200 and 400 mg/kg) in rats for 28 days	Anti-insulin, anti-lipid, anti-obesity, reduced blood pressure and anti-inflammatory effect	Moderate
Tan et al. (2011) (Tan et al., 2011)	China	*In vivo*	The effect of Chinese herbal extracts on metabolic syndrome	N = 36	Intake of 4 g/kg for 4 weeks	Reduction of visceral fat, cholesterol and triglycerides	Moderate
Chen et al. (2011) (Chen et al., 2011)	The U.S	*In vivo*	Effects of Green Tea on Metabolic Syndrome	N = 118	Receiving 10 cups of green tea (2 g tea leaves per cup) per day for 17 weeks	Reducing lipid absorption and reduced levels of inflammatory cytokines	Moderate
Chen et al. (2021) (Chen et al., 2021)	China	*In vivo*	Effect of *Ligustrum* robustum (Roxb.) blume extract on Metabolic Syndrome	-	Receiving 200 mg/kg/d for 16 weeks	Preventing gut microbiota dysbiosis	Moderate
Kasabri et al. (2014) (Kasabri et al., 2014)	Jordan	*In vivo*	Effect of *Salvia officinalis* on Metabolic Syndrome	-	125, 250, 500, 1,000 and 2000 μg/mL	Antidiabesity	Low
Mayer et al. (2022) (Mayer et al., 2022)	France	*In vivo*	The effect of the Marine Microalga *Diacronema lutheri* on Metabolic Syndrome	N = 18	Daily intake of 24 mg	Preventing of weight gain and improving in lipid and glucose homeostasis	Moderate
De Martin et al. (2018) (De Martin et al., 2018)	Italy	Experimental	The effect of two types of *Phaeophyceae* on metabolic syndrome	N = 50	Daily administration of 3 capsules containing algae (237.5 mg Ascophyllum nodosum, 12.5 mg Fucus vesiculosus and 7.5 μg chromium) for 6 months	Reduction of waist circumference, glucose level, blood insulin	Moderate
Hermans et al. (2020) (Hermans et al., 2020)	Belgium	Observational	Effect of a Combination of Olive (*Olea europea L.*) Leaf and Fruit Extracts on Metabolic Syndrome	N = 145	Receiving 2 capsules/daily (each capsule contains 167 mg of Olea europea leaf dry extract and 53 mg of Olea europea L. fruit dry extract)	Reducing triglycerides, fasting blood glucose, waist circumference, hypertension and increasing high-density lipoprotein cholesterol (HDL-C)	High
Li et al. (2020) (Li et al., 2020)	China	*In vivo*	Effect of *Eriobotrya japonica* leaf on metabolic syndrome	-	Receiving 200 mg/kg for 12 weeks	Reducing body weight, relative liver weight, relative visceral adipose weight, cholesterol, triglycerides, low-density lipoprotein cholesterol, hepatic total cholesterol, and hepatic triglycerides	Moderate
Benkhaled et al. (2020) (Benkhaled et al., 2022)	Algeria	*In vivo*	effects of aqueous leaf extract of *Limoniastrum* guyonianum on metabolic syndrome	N = 42	Receiving 100, 200 and 300 mg/kg b.w./day for 9 weeks	Hypoglycemic, hypolipidemic, antioxidant and renoprotective	Moderate
Basu et al. (2013) (Basu et al., 2013)	The U. S	Clinical trial	The effect of green tea on metabolic syndrome	N = 35	Daily intake of 4 cups of green tea or 2 capsules of green tea extract for 8 weeks	Antioxidant activity	High
Nagata et al. (2012) (Nagata et al., 2012)	Japan	Clinical trial	The effect of KBG^2^ on endothelial function in patients with metabolic syndrome	N = 92	Use of 2 g of KBG, three times a day after meals for 4 weeks	Improving endothelial function in patients with metabolic syndrome factors	High
Gurrola-Di´az (2010) (Gurrola-Dlaz et al., 2010)	Mexico	Clinical trial	The effect of *Hibiscus sabdariffa* extract on metabolic syndrome	N = 124	Daily intake of 100 mg of plant extract for 1 month	Reducing glucose and total cholesterol levels, improving insulin resistance, reducing triglycerides, blood pressure, and dyslipidemia	Moderate
Verhoeven et al. (2015) (Verhoeven et al., 2015)	Belgium	Clinical trial	The effect of red yeast rice and *Olea europaea* (olive) extract on metabolic syndrome	N = 50	Daily intake of a product containing 10.82 mg of monaculin and 9.32 mg of hydroxytyrosol, for 8 weeks	Lowering cholesterol (LDL) and blood pressure	High
Vitamins							
Ferreira et al. (2020) (Ferreira et al., 2020)	Brazil	Clinical trial	The effect of vitamin D3 on metabolic syndrome in postmenopausal women	N = 160	Intake of 1,000 units of vitamin D3 per day for 9 months	Decrease in triglycerides and insulin resistance and reduction in the risk of hypertriglyceridemia and hyperglycemia	High
Farag et al. (2019) (Farag et al., 2019)	Iraq	Clinical trial	The effect of vitamin C on metabolic syndrome	N = 120	Daily intake of 500 mg for 12 weeks	Reduced BMI	High
Wenclewska et al. (2019) (Wenclewska et al., 2019)	Poland	Clinical trial	The effect of vitamin D on metabolic syndrome	N = 92	Daily intake of 2000 units of vitamin D for 3 months	Reduced DNA damage, oxidative stress, and HbA1c	High
Ahmadi et al. (2014) (Ahmadi et al., 2014)	Iran	Clinical trial	The effects of vitamin E and omega-3 on metabolic syndrome	N = 90	Daily intake of 400 units of vitamin E or 2.4 g of omega-3 for 8 weeks	Improving endothelial function using omega-3, lack of effect of vitamin E on endothelial function	High
Farag et al. (2018) (Farag et al., 2018)	Iran	Clinical trial	effects of vitamin D and vitamin C supplementations on metabolic syndrome	N = 180	Receiving 500 mg/day vitamin C or 2000 IU/day vitamin D for 12 weeks	Improvements fasting plasma glucose, total cholesterol, low-density lipoprotein cholesterol and blood pressure, waist circumference, triglyceride, and high-density lipoprotein	High
Salekzamani et al. (2016) (Salekzamani et al., 2016)	Iran	Clinical trial	Effect of high-dose vitamin D supplementation on metabolic syndrome	N = 80	Receiving 50,000 IU vitamin D weekly for 16 weeks	Decreasing triglyceride, but did not have any beneficial effects on other cardiometabolic risk factors	High
Salekzamani et al. (2017) (Salekzamani et al., 2017)	Iran	Clinical trial	Effect of vitamin D supplementation on metabolic syndrome	N = 80	Receiving 50,000 IU vitamin D weekly for 16 weeks	Improvement some proatherogenic inflammatory markers, but no changes of high sensitivity C-reactive protein and carotid intima media thickness	High
Erbas et al. (2014) (Erbaş et al., 2014)	Turkey	*In vivo*	The effect of vitamin D on metabolic syndrome	N = 24	Intake of 0.3 μg/kg/day for 2 weeks	Anti-inflammatory, antioxidant activities	Moderate
Bilbis et al. (2012) (Bilbis et al., 2012)	Nigeria	*In vivo*	The effect of vitamins A, C and E on the treatment of metabolic syndrome in mice	N = 30	Daily intake of 100 mg/kg of vitamin C	Reducing blood pressure, serum total cholesterol, triglycerides, low and very low-density lipoprotein cholesterol, and increasing high-density lipoprotein cholesterol and antioxidant activity	Moderate
					6 mg/kg vitamin A, 60 mg/kg vitamin E for 4 weeks		
Mazur-Bialy & Poche´c (2016) (Mazur-Bialy and Pocheć, 2016)	Poland	*In vivo*	The effect of vitamin B2 on metabolic syndrome	-	Receiving 10.4–1,000 nM	Reducing the conditions associated with the mild inflammation linked to obesity	Moderate
Mostafa et al. (2016) (Mostafa et al., 2016)	Egypt	*In vivo*	The effect of vitamin D on metabolic syndrome	N = 50	Receiving 6 ng/kg SC vitamin D daily for 8 weeks	Improve hypertension, metabolic and structural abnormalities	Moderate
Manning et al. (2013) (Manning et al., 2013)	Australia	Clinical trial	The effect of lipoic acid and vitamin E on metabolic syndrome	N = 151	Receiving 100 IU/day vitamin E or 600 mg/day lipoic acid for 12 months	Reducing plasma NEFA^3^, But does not change insulin or glucose levels	High
Matsumoto et al. (2011) (Matsumoto et al., 2011)	Japan	*In vivo*	Effects of Zn(II) complex with vitamins C and U, and carnitine on metabolic syndrome	N = 30	Receiving 40 mg zinc kg1 body weight over the period of 9–13 weeks	Reducing the visceral adipose tissues content and/or improvement in blood fluidity	Moderate
Minerals							
Kelishadi et al. (2010) (Kelishadi et al., 2010)	Iran	Clinical trial	The effect of zinc on metabolic syndrome	N = 60	Daily intake of 20 mg of zinc for 8 weeks	Reduction of total cholesterol and LDL, insulin resistance, and BMI	High
Dietary Supplements							
Usui et al. (2013) (Usui et al., 2013)	Japan	Clinical trial	The effect of natural supplement of S-equol on metabolic syndrome	N = 54	Daily intake of 10 mg of S-equol for 12 weeks	Reduction of HbA1c, serum low-density lipoprotein cholesterol level and CAVI^4^ score	High
McPherson et al. (2016) (McPherson et al., 2016)	Australia	*In vivo*	Effect of micronutrient supplementation on metabolic syndrome	N = 20	Receiving a diet containing vitamin C, vitamin E, folate, lycopene, zinc, selenium and green tea extract for 17 weeks	Preventing early growth restriction, fat accumulation and dyslipidaemia	Low
Akrami et al. (2018) (Ak et al., 2018)	Iran	Clinical trial	effects of flaxseed (*Inum usitatissimum*) oil and sunflower seed oil on metabolic syndrome	N = 60	Receiving 25 mL/d SO or 25 mL/d FO for 7 weeks	Reducing total cholesterol, low-density lipoprotein cholesterol, and triglyceride levels, systolic and diastolic BP, weight, waist circumference	High
Sanchez-Rodriguez et al. (2018) (Sanchez-Rodriguez et al., 2018)	Spain	Clinical Trials	Effects of Virgin Olive Oils on Metabolic Syndrome	N = 58	Daily intake 30 mL for 3 weeks	Improving high density lipoprotein cholesterol (HDLc)	High
Pedersen et al. (2010) (Pedersen et al., 2010)	Denmark	Clinical Trials	Effects of fish oil supplementation on metabolic syndrome	N = 78	Receiving 1.5 g/day fish oil for 16 weeks	Improving blood pressure	High
Ruyvaran et al. (2022) (Ruyvaran et al., 2022)	Iran	Clinical Trials	The effect of Safflower (*Carthamus tinctorius L*.) oil on metabolic syndrome	N = 67	Daily intake 8 gr Safflower oil for 12 weeks	Improving abdominal obesity, blood pressure, and insulin resistance	High
Nimrouzi et al. (2020) (Nimrouzi et al., 2020)	Iran	*In vivo*	The effect of Oil and extract of safflower seed on metabolic syndrome	N = 80	Receiving different doses of oil and extract daily for 16 weeks	Antioxidant and anti-inflammatory	Moderate
Pilar et al. (2017) (Pilar et al., 2017)	Brazil	*In vivo*	The effect of Flaxseed Oil and Flaxseed Lignan Secoisolariciresinol Diglucoside on Metabolic Syndrome	N = 48	Receiving 1 mL/kg body weight FO or 20 mg/kg body weight SDG for 30 days	Reducing oxidative stress	Moderate
Olid et al. (2023) (Olid et al., 2023)	Spain	*In vivo*	Effect of extra virgin olive oil compared to butter on metabolic syndrome	N = 35	Receiving olive oil or butter for 12 weeks	Antimicrobial activity to keep blood pressure low	Moderate
Ram´rez-Higuera et al. (2019) (Ramírez-Higuera et al., 2020)	Mexico	*In vivo*	Sterculic Oil on Metabolic Syndrome	N = 24	Receiving 0.06 g of SO emulsified with 3% Tween 20 in water for 8 weeks	Improvement blood pressure, insulin resistance, serum glucose and triglycerides, steatosis, and adiposity	Moderate
Shen et al. (2020) (Shen et al., 2020)	The U.S	*In vivo*	Effect of Milk thistle (*Silybum marianum*) seed cold press oil on metabolic syndrome	N = 15	Receiving 0.1% oil/50 gm body weight/day for 8 weeks	Reducing obesity, fasting hyperglycemia, hypertension, and induced markers of mitochondrial fusion and browning of white adipose	Moderate
Barrios-Ramos et al. (2012) (Barrios-Ramos et al., 2012)	Mexico	*In vivo*	Effect of cocoa, soy, oats and fish oil on metabolic syndrome	N = 96	Receiving the mix of cocoa + soy + oats + fish oil for 14 weeks	Reducing levels of triglycerides, glucose, blood pressure and total cholesterol	Moderate
Mert et al. (2022) (Mert et al., 2022)	Turkey	*In vivo*	The effect of evening primrose oil (Oenothera biennis) on metabolic syndrome	N = 40	Daily Intake 0.1 mL primrose oil for 57 days	Reducing oxidative stress, increasing adiponectin levels and insulin sensitivity, anti-inflammatory, regulating dyslipidemia and systolic blood pressure	High
Padiya et al. (2011) (Padiya et al., 2011)	India	*In vivo*	Effect of Garlic on metabolic syndromes	N = 21	Receiving 250 mg/kg/day for 8 weeks	Reducing serum glucose, insulin, triglyceride and uric acid levels, insulin resistance	Moderate
Pérez-Torres et al. (2016) (Pérez-Torres et al., 2016)	Mexico	*In vivo*	The effect of garlic on metabolic syndrome in rats	N = 16	Intake of 125 mg of extract every 12 h	Reducing cholesterol, improving heart function and reducing coronary artery resistance	Moderate
Hi and Endang (2020) (Hi and Endang, 2020)	Indonesia	Observational	The Effect of Black Seed Oil on metabolic syndrome	N = 62	Intake BSO with a dose of 3 mL/day for 20 days	No significant difference in both groups test in the BMI, blood serum glucose, blood pressure and cholesterol levels	High
Al-Okbi et al. (2014) (Al-Okbi et al., 2014)	Egypt	*In vivo*	Effect of Clove Oil and Eugenol Microemulsions on Metabolic Syndrome	N = 30	Receiving 40 mg CO/kg rat body weight, or 31 mg eugenol/kg rat body weight	Improvement on inflammatory fatty liver (steatohepatitis) and dyslipidemia	Moderate

1 epiogallocatechin-3-gallate.

2 - keishibukuryogan (KBG; Guizhi-Fuling-Wan) (Dried herbal powder consisting of Amomi Cortex, Paeoniae Radix, Moutan Cortex, Persicae Semen and Hoelen mixed with honey.).

3 nonesterified fatty acid.

4 - Cardio-ankle vascular index.

### 2.4 Quality assessment of articles

The Mixed Methods Appraisal Tool (MMAT) was used to evaluate the quality of the studies (Pace et al., 2012). Each section of the tool is categorized based on the research design employed. This tool is valuable for assessing the appropriateness of a study’s objective, methods, study design, data collection, study selection, data analysis, presentation of findings, discussion, and conclusion(s). The quality of the articles and their inclusion after data extraction are determined by reviewing these aspects. Articles in each domain are assessed for quality using a percentage scale ranging from 25% (indicating that only one criterion is met) to 100% (indicating that all criteria are met). In this study, articles scoring below 25% are considered low quality, while those scoring above 80% are considered high quality (Pace et al., 2012; Madlabana et al., 2020). Based on the findings of the current study, the evaluation of article quality yielded an average score of 67.5%.

## 3 Results

The analysis of Table 1 data presents a comprehensive view of the impact of natural products on metabolic syndrome, reflecting a broad spectrum of research methodologies including clinical trials, *in vitro* and *in vivo* studies, and observational analyses. This diversity underscores the multifaceted approach in investigating the potential of natural interventions. Categorization by type of natural product reveals a predominant focus on plant-based interventions, signalling a notable scientific interest in exploring their efficacy in addressing metabolic syndrome. However, amidst the majority of studies demonstrating high to moderate quality, one *in vivo* article stands out for its notably low quality. Nonetheless, the collective outcomes suggest promising benefits associated with natural products, spanning various metabolic parameters such as blood pressure, glucose levels, lipid profile, and markers of obesity. These findings underscore the potential utility of natural interventions in managing the complexities of metabolic syndrome.

The predominance of clinical trials and *in vivo* studies in Table 1 underscores a comprehensive approach to investigating the therapeutic potential of natural products for managing metabolic syndrome. Clinical trials, being directly relevant to human health outcomes, provide crucial evidence regarding the efficacy and safety of interventions in real-world settings, reflecting a commitment to evidence-based medicine. Additionally, the inclusion of *in vivo* studies offers valuable insights into the underlying biological mechanisms of natural interventions, informing the design and interpretation of clinical trials. This diversity of research approaches not only contributes to a nuanced understanding of the efficacy and safety profiles of natural products but also highlights their translational potential from preclinical research to clinical practice.

### 3.1 Herbs

Upon evaluating the results of the studies, it becomes evident that herbs play a significant role in addressing various aspects of metabolic syndrome. A wide array of herbs have demonstrated the capacity to directly influence key metabolic parameters such as blood pressure, blood glucose levels, lipid profiles, obesity, and cholesterol and triglyceride levels (Nikaein et al., 2016; Seong et al., 2021; Amin et al., 2015; Belcaro et al., 2013; Devaraj et al., 2013; Najmi et al., 2013; Mansouri et al., 2012; Shah et al., 2012; Basu et al., 2010; Gupta Jain et al., 2017; Mopuri et al., 2018; Cicolari et al., 2020; Chae et al., 2022; Dobhal et al., 2022; Jakovljevic et al., 2018; Reshidan et al., 2019; Verhoeven et al., 2015; Gurrola-Dlaz et al., 2010; Benkhaled et al., 2022; Li et al., 2020; Hermans et al., 2020; De Martin et al., 2018; Mayer et al., 2022; Kasabri et al., 2014; Tan et al., 2011; Kuate et al., 2015; Kho et al., 2016). However, while the breadth of herbs studied is extensive, evaluations reveal variations in their efficacy and mechanisms of action.

Several plants exhibit promising effects through mechanisms such as antibacterial activity (Ghitea et al., 2021), antioxidant properties (Basu et al., 2013; Lopes et al., 2014), and the presence of bioactive compounds (Jakubczyk et al., 2021). These mechanisms contribute to the prevention of inflammation and insulin resistance (Kulabas et al., 2018), reduction of fat mass, and even serve as sources of essential amino acids and prebiotics (du Preez et al., 2021). Additionally, certain plants show potential in improving kidney function (Owis et al., 2017), regulating glucose and fatty acid metabolism (Reshidan et al., 2019), and balancing lipoprotein secretion and energy homeostasis (Singh et al., 2017). Furthermore, some plants demonstrate the ability to restore abnormal blood glucose regulation and rebalance intestinal microbiota (Wang et al., 2020), thus presenting multifaceted approaches to addressing metabolic syndrome.

However, evaluations also reveal complexities and contradictions within the findings. For instance, while the majority of plants studied exhibit beneficial effects, the use of green *Elettaria cardamomum* in one particular *in vivo* study led to unexpected outcomes. Contrary to expectations, green cardamom was found to exacerbate obesity, impair liver function, and adversely affect cardiovascular structure and function (Bhaswant et al., 2015). On the other hand, one clinical trial concluded that *Ganoderma lucidum* had no significant impact on hyperglycemia and cardiovascular risk factors (Klupp et al., 2016). Similarly, another clinical trial found that *Cuminum cyminum L* only decreased diastolic blood pressure without affecting other parameters of metabolic syndrome (Morovati et al., 2019). Additionally, the results of a clinical trial study indicated that *Nigella Sativa* failed to yield no significant differences between the two study groups regarding BMI, blood serum glucose, blood pressure, weight, WC and cholesterol levels (Mohtashami, 2019).

### 3.2 Vitamins and minerals

The results gleaned from studies investigating the impact of vitamins and minerals on metabolic syndrome and its components shed light on the potential benefits and limitations of various vitamin interventions. Specifically, vitamins C, D, E, A, B2, and U and zinc have demonstrated efficacy in influencing key parameters associated with metabolic syndrome, including blood pressure, blood glucose levels, lipid profiles, obesity, cholesterol, and triglycerides (Kelishadi et al., 2010; Farag et al., 2018; Farag et al., 2019; Ferreira et al., 2020). Additionally, these vitamins have shown promise in reducing DNA damage and oxidative stress (Wenclewska et al., 2019), as well as inflammatory markers (Erbaş et al., 2014; Mazur-Bialy and Pocheć, 2016; Salekzamani et al., 2017), while also decreasing plasma non-esterified fatty acids (NEFA), thereby contributing to the improvement of metabolic syndrome. However, critical evaluation of the findings reveals inconsistencies and challenges in translating these results into clinical practice. For instance, two clinical trial studies reported no significant effect of vitamin E on endothelial function, insulin levels, or glucose levels, highlighting a discrepancy between theoretical benefits and observed outcomes (Manning et al., 2013; Ahmadi et al., 2014). Similarly, two other clinical trials found that vitamin D supplementation failed to yield beneficial effects on cardiometabolic risk factors, high-sensitivity C-reactive protein levels, and carotid intima-media thickness (Salekzamani et al., 2016; Salekzamani et al., 2017).

### 3.3 Dietary supplements

Dietary supplements represent another category of natural products that have shown promise in addressing various aspects of metabolic syndrome, as indicated by the findings of studies included in this review. These supplements have demonstrated the ability to mitigate early growth restriction and inhibit fat accumulation by effectively modulating key metabolic parameters such as blood pressure, blood glucose levels, dyslipidemia, obesity, cholesterol, and triglycerides (Pedersen et al., 2010; Barrios-Ramos et al., 2012; Usui et al., 2013; McPherson et al., 2016; Ak et al., 2018; Sanchez-Rodriguez et al., 2018; Ramírez-Higuera et al., 2020; Shen et al., 2020; Ruyvaran et al., 2022). Additionally, they have been associated with improvements in insulin resistance (Mert et al., 2022), exerting antioxidant and anti-inflammatory effects (Nimrouzi et al., 2020), reducing oxidative stress (Pilar et al., 2017), decreasing coronary artery resistance (Pérez-Torres et al., 2016), and ameliorating inflammatory fatty liver conditions such as steatohepatitis (Al-Okbi et al., 2014). Noteworthy among these supplements are nutrients, garlic, as well as vegetable and animal oils. Despite various findings on the effectiveness of nutritional supplements for metabolic syndrome, an observational study yielded inconclusive results. It showed that black seed exhibited no significant difference between the two studied groups in terms of BMI, serum glucose, blood pressure, weight, waist circumference, and cholesterol levels (Hi and Endang, 2020).

## 4 Discussion

This scoping review was conducted to investigate the influence of natural products on metabolic syndrome. Based on the findings from the reviewed studies, we obtained a wide range of natural products, including herbs, vitamins, minerals and dietary supplements that affected the treatment and prevention of metabolic syndrome. These products have effects on blood pressure, blood sugar, obesity, waist circumference fat, and abnormal cholesterol or triglyceride levels.

The findings of this research show that plants play a role in improving metabolic syndrome by influencing blood glucose levels, lipid profile, obesity, cholesterol, and triglyceride levels. Various studies have identified a wide range of plants for this purpose. For instance, a review study by Pérez-Muñoz et al. (2022) (Pérez-Muñoz et al., 2022) demonstrated that the Eryngium plant reduces LDL and HDL levels, improves glucose metabolism, and controls dyslipidemia and blood glucose. In another study, Akber Aisa et al. (2019) (Aisa et al., 2019) found that Cicer arietinum L modulates glycemic response and regulates dyslipidemia. A systematic review and meta-analysis by García-Muñoz et al. (2023) (García-Muñoz et al., 2023) reported the effectiveness of Hibiscus sabdariffa in weight loss, reduction of fat tissue, lowering blood pressure, and improving lipid profiles. Apart from studies directly emphasizing the effects of plants on the main components of metabolic syndrome, some studies have also shown that plants can improve metabolic syndrome through antibacterial activities, antioxidant properties, anti-inflammatory activities, and reduction of insulin resistance. One such plant is spirulina, which possesses anti-inflammatory properties. By reducing inflammation, this herb can help improve vascular function and blood pressure, thereby positively impacting metabolic health (Yousefi et al., 2018).

Green tea and turmeric also possess antioxidant and anti-inflammatory properties. In metabolic syndrome, chronic low-grade inflammation and oxidative stress can contribute to insulin resistance and impaired glucose metabolism. Therefore, these herbs can help mitigate these effects, leading to improved insulin sensitivity and lower blood sugar levels (Basu et al., 2013; Amin et al., 2015). Additionally, green tea contains polyphenols such as epigallocatechin gallate (EGCG), which have demonstrated the potential to increase fat oxidation and thermogenesis, aiding in weight management (Basu et al., 2013; Wong et al., 2020). Another herb that can assist in regulating blood sugar levels is cinnamon, which can impact fat storage and metabolism by regulating blood sugar levels (Mollazadeh and Hosseinzadeh, 2016).

Contrary to the aforementioned findings, some studies present conflicting results and suggest that herbs may not have a significant impact on metabolic syndrome and its components. For instance, one study revealed that *G. lucidum* does not exhibit a significant effect on blood sugar and cardiovascular risk factors (Klupp et al., 2016). Similarly, another study found that *Nigella Sativa* did not yield statistically significant differences between the two study groups regarding BMI, blood serum glucose, blood pressure, weight, waist circumference, and cholesterol levels (Mohtashami, 2019). Additionally, according to the results of another study, *E. cardamomum* was associated with obesity, liver dysfunction, and adverse effects on cardiovascular structure and function (Bhaswant et al., 2015). Several factors could contribute to these discrepancies in results. Variations in study design, such as sample size and duration, as well as the diversity of bioactive compounds in plants, may be among the reasons. Demographic differences, dosage variations, and confounding variables such as diet and lifestyle could also play a role. Furthermore, publication bias, study quality, and the inherent complexity of metabolic syndrome itself contribute to variability in outcomes. Ultimately, these findings underscore the necessity for further research, particularly clinical trials, to elucidate the precise mechanisms and potential adverse effects associated with specific herbal interventions for metabolic syndrome.

It is important to note that the mechanisms of action for natural products can vary widely, and not all natural products have been extensively studied in the context of metabolic syndrome. Additionally, individual responses to natural products can vary, and their effects may depend on factors such as dosage, duration of use, and specific characteristics of a person with metabolic syndrome. Therefore, it is necessary to conduct research, particularly clinical studies, on the effect of natural products on the components of metabolic syndrome.

Another finding of the present study suggests that vitamins and minerals can play an effective role in managing metabolic syndrome and its components. For instance, the study by Melguizo-Rodríguez et al. (2021) (Melguizo-Rodríguez et al., 2021) demonstrated that vitamin D supplementation improves lipid profile, insulin resistance, hyperglycemia, obesity, and high blood pressure. Similarly, Moukayed & Grant et al. (2019) (Moukayed and Grant, 2019)discovered that vitamin D possesses anti-inflammatory properties, inhibits primary adipogenesis, enhances glucose absorption, mitigates hyperleptinemia, ameliorates insulin resistance, and reduces high blood pressure. Wong et al. (2020) (Wong et al., 2020) confirmed the antioxidant and anti-inflammatory properties of vitamin C.

However, there are also several studies that contradict the positive effects of vitamins and nutrients on metabolic syndrome. Ahmadi et al. (2014) (Ahmadi et al., 2014) and Manning et al. (2015) (Manning et al., 2013) reported no significant effects of vitamin E on endothelial function, insulin levels, or glucose levels. Similarly, studies by Salekzamani et al. (2017) (Salekzamani et al., 2017) and Salekzamani et al. (2016) (Salekzamani et al., 2016) indicated that vitamin D supplementation failed to yield beneficial effects on cardiometabolic risk factors, high-sensitivity C-reactive protein levels, and carotid intima-media thickness. AlAnouti et al.’s study (2020) (AlAnouti et al., 2020)also demonstrated the limited impact of vitamin D on improving dyslipidemia. Furthermore, Panchal et al. (2017) (Panchal et al., 2017) suggested conflicting results regarding the effect of these micronutrients on metabolic syndrome.

The inconsistency in findings regarding the effect of vitamins on metabolic syndrome can be attributed to several factors. Variations in study design, such as sample size, duration of intervention, and methods used to measure outcomes, can lead to conflicting results. Studies with small sample sizes or short durations may fail to capture the full effects of vitamins and nutrients on components of the metabolic syndrome. Additionally, differences in study population characteristics, including age, gender, ethnicity, and baseline health status, can influence the response to vitamin and nutrient supplementation. What proves effective in one population may not yield the same results in another.

Furthermore, vitamins may interact with other nutrients or medications, potentially influencing their effects on metabolic syndrome, and these interactions can differ between studies. Conversely, the dose and duration of vitamin and nutrient supplementation can significantly impact their effectiveness. Therefore, it is essential to design studies with optimal doses and longer durations to observe the significant effects of vitamins and nutrients on the components of metabolic syndrome.

The results of the present study also confirmed the effect of food supplements, including nutrients and vegetable and animal oils, on the components of metabolic syndrome. Vegetable and animal oils exert their effect on metabolic syndrome through a combination of factors. The composition of their fatty acids plays an essential role. Oils rich in unsaturated fats, such as olive oil and certain vegetable oils, as well as those containing omega-3 fatty acids, such as flaxseed and fish oil, help improve lipid profiles and reduce inflammation, and are beneficial for people with metabolic syndrome. The results of the studies by Nimrouzi et al. (2020) (Nimrouzi et al., 2020), Mert et al. (2022) (Mert et al., 2022), and Al-Okbi et al. (2014) (Al-Okbi et al., 2014) confirmed these findings. Additionally, Olid et al. (2023) (Olid et al., 2023) stated that olive oil has antimicrobial activity to keep blood pressure low.

The results of a study showed that omega-3 fatty acids, which are found in some natural products such as flax seeds, have demonstrated the ability to reduce the levels of triglycerides and cholesterol by decreasing the production of triglycerides in the liver and increasing the clearance of triglycerides from the bloodstream (Yang et al., 2020). Additionally, flaxseed oil exhibited the ability to reduce oxidative stress in a study (Mohammadifard et al., 2021).

Garlic is another food supplement that contains allicin, and by increasing the production of nitric oxide in the blood vessels, it induces vasodilation. This widening of blood vessels can lead to a decrease in resistance to blood flow and subsequently result in a decrease in blood pressure (Al Disi et al., 2016). However, the results of Hi et al.’s study (2020) (Hi and Endang, 2020) showed that black seed oil did not cause significant differences in BMI, blood glucose, blood pressure, and cholesterol levels between the two groups. Furthermore, the results of Tamtaji et al. (2020) (Tamtaji et al., 2020) indicated that flaxseed oil had no effect on certain inflammatory factors and other biomarkers of oxidative stress. Similarly, Pastor et al. (2021) (Pastor et al., 2021) demonstrated that olive oil has no effect on metabolic syndrome.

Biological variability among participants, genetic factors, publication bias, different study designs, varied doses, and the overlap of some foods with dietary supplements may have influenced the study results, highlighting the importance of well-designed randomized controlled trials. Systematic reviews are essential to elucidate the true effects of dietary supplements on metabolic syndrome and to provide evidence-based recommendations.

The strength of the present study lies in comprehensive exploration of natural products in relation to their potential impact on metabolic syndrome and its components. This broad perspective adds to the existing body of knowledge and provides valuable insights into the potential benefits of these products for people with metabolic syndrome. However, this study also had limitations. For instance, despite the researchers’ efforts to obtain the full text of the articles, this was not achieved in the case of some articles, and as a result, some related articles were excluded from the research. Additionally, while eligible articles were identified and reviewed, it is possible that some unpublished studies may have been missed. Utilizing specific entry criteria, such as excluding articles published before 2010 in this study, may result in the omission of relevant articles. However, the researchers in this particular case adopted this criterion to enhancing the organization of the articles. Also, Utilizing a particular search strategy in articles may result in the omission of related articles.

## 5 Conclusion

This study underscores the considerable potential of natural products, including herbs, vitamins, minerals, and dietary supplements, in effectively managing metabolic syndrome. These interventions offer promising avenues for addressing various aspects of metabolic syndrome through diverse mechanisms of action. Among natural products, the study found that plants have been the subject of more research compared to other categories. While both positive and negative effects have been observed across all categories of natural products, there appears to be more uncertainty surrounding the effects of vitamins. This suggests that the effects of vitamins are often unclear and may differ depending on the specific circumstances. However, the study acknowledges the need for further research to validate these findings and determine the optimal dosage and duration of supplementation. Long-term randomized controlled trials with larger sample sizes are particularly necessary to provide more conclusive evidence regarding the efficacy and safety of natural products in managing metabolic syndrome.
